# Nonhemolytic, Nonmotile Gram-Positive Rods Indicative of *Bacillus anthracis*

**DOI:** 10.3201/eid0908.030205

**Published:** 2003-08

**Authors:** Elie G. Dib, Samar A. Dib, Dany A. Korkmaz, Neville K. Mobarakai, Jordan B. Glaser

**Affiliations:** * Staten Island University Hospital, Staten Island, New York, USA

**Keywords:** *Bacillu*s *megaterium*, anthrax, Bacillus, ciprofloxacin

## Abstract

We report a 40-year-old female patient who was admitted to the hospital because of a left ovarian mass torsion. A nonhemolytic, nonmotile *Bacillus*, suspicious of *Bacillus anthracis,* was isolated from a blood culture. We discuss the evaluation that led to the final identification of the bacterium as *B. megaterium*.

*Bacillus* represents a genus of ubiquitous gram-positive bacteria. The species are used in many medical, pharmaceutical, agricultural, and industrial processes, including those for making antibiotics and insecticides (*1*–*4*). Even the anthrax toxin is being evaluated as a choice for tumor cell surface targeting in chemoresistant neoplasms (*5*).

Although most species are harmless, two are medically significant: *Bacillus anthracis* and *B. cereus*. *B. anthracis* causes anthrax in its cutaneous, pulmonary (inhalational), and intestinal forms. *B. cereus* causes two distinct food poisoning syndromes, a rapid-onset emetic syndrome characterized by nausea and vomiting and a slower onset diarrheal syndrome.

*Bacillus* are often isolated on blood culture and usually represent blood culture contamination. For example, *Bacillus* species pseudobacteremia has been traced to contaminated gloves used in collection of blood from patients (*6*). In immunocompromised hosts, a blood culture growing *Bacillus* species should be evaluated carefully. Rarely, these species cause important clinical diseases such as bacteremia, sepsis, meningitis, pneumonia, empyema, ophthalmitis, osteomyelitis, endocarditis, soft tissue infection, and intravascular catheter-acquired sepsis.

Pseudotumour of the lung has been reported as the cause of infection with *B. sphaericus* (*7*). Endocarditis has been reported to be caused by *B. subtilis* (*8*). An outbreak of *Bacillus* species in a cancer hospital in Brazil was reported (*9*) and was strongly associated with use of calcium gluconate solution and central venous lines. The outbreak was controlled by stopping use of the implicated calcium gluconate vials.

## Case Study

A 40-year-old woman, with no significant medical history, was seen at the emergency room because of worsening left lower quadrant abdominal pain. The pain, which had started a few days previously, was constant, localized to the left lower quadrant of the abdomen, and described as dull and moderately to severely intense. The pain was not related to meals or bowel movements and was not accentuated or relieved by any specific position. The patient noted constipation but had no nausea or vomiting. No rectal bleed or melena occurred, and she reported no urinary symptoms or vaginal discharge. Her last normal menstrual period was 8 days before. She felt warm but did not check her temperature and did not experience chills. Her primary medical physician prescribed ciprofloxacin 500 mg orally twice a day for the presumptive diagnosis of colitis. She took the antibiotic for 2 days without improvement.

In the emergency room, the patient was afebrile and hemodynamically stable. The physical examination showed tenderness on palpation of the left lower quadrant of the abdomen with minimal rebound tenderness. The pelvic examination showed left adnexal tenderness with a possible mass. Results of urinalysis and a urine pregnancy test were negative. No leukocytosis was noted. A pelvic ultrasonograph showed a left ovarian complex mass measuring 14 cm x 9 cm x 6 cm as well as a moderate amount of free fluid in the cul-de-sac. The study suggested left ovarian mass torsion. A laparascopic resection was performed successfully. The patient received intravenous cefazolin perioperatively. The final pathology report showed a mature teratoma of the left ovary featuring dermoid cyst, respiratory anlaga, and struma ovarii. The patient improved and was discharged 2 days after surgery.

One day later, a blood culture, drawn in the emergency room, grew nonhemolytic, nonmotile gram-positive rods. *B. anthracis* was suspected. The blood culture was reported to the New York City Department of Health. The patient was called for reassessment at the hospital. She was afebrile, and her only complaint was mild low back pain. She had mild dry cough, but results of a chest roentgenogram were unremarkable. She was started on intravenous clindamycin, ciprofloxacin, and rifampin. Two days later, the New York City Department of Health reported the following: results of the direct fluorescent-antibody (DFA) assay, using fluorescein-labeled monoclonal antibodies specific to the *B. anthracis* capsule (CAP-DFA) antigens, were positive; results of the DFA assay, using fluorescein-labeled monoclonal antibodies specific to the *B. anthracis* cell wall (CW-DFA) were negative; and the *Bacillus* isolated was not lysed by the γ phage.

The organism was confirmed to be *Bacillus* non-*anthracis*. Based on its characteristics, it was classified as *B. megaterium*. The information was disclosed to the patient, and the intravenous antibiotic therapy was discontinued. The patient’s initial dry cough had resolved, and she had no evidence of any cutaneous, respiratory, or neurologic sign of disease. She was counseled about any potential side effects of the antibiotic therapy she had received, otherwise reassured, and then discharged.

## Conclusions

Presumptive identification of *B. anthracis* in a hospital laboratory is based on the direct Gram-stained smear of a skin lesion, cerebrospinal fluid, or blood showing encapsulated, broad, gram-positive bacilli. Indicators of growth apparent on cultures are also factors. *B.* *anthracis* is nonmotile and nonhemolytic on sheep’s-blood agar. In vitro it grows as long chains, but in the host *B. anthracis* appears as single organisms or chains of two or three bacilli. The organism forms mucoid colonies and exhibits a prominent capsule when grown on nutrient agar containing 0.7% sodium bicarbonate in the presence of 5% to 20% carbon dioxide (*10*).

The only nonmotile *Bacillus* are *B. anthracis* and *B. cereus* subsp. *mycoides*. Some other *Bacillus* species show variable motility and may often be nonmotile. These species include *B. megaterium*, *B. firmus*, and *B. circulans*. At the community laboratory level, once the *Bacillus* colonies are identified as catalase-positive, nonhemolytic, nonmotile gram-positive rods, the organism should be packaged properly and transported to a state or county public health laboratory for confirmation (*11*).

Confirmatory diagnostic tests are performed at the Laboratory Response Network for Bioterrorism (LRN) (*11*), which consists of laboratories at four levels (*12*–*14*). Laboratories at the community level, considered level A, should recognize the clues to a suspicious agent and package the agent for transfer to the next higher level laboratory.

Level-B laboratories often include the state and county public health laboratories. Criteria for confirming *B. anthracis* at this level include susceptibility to lysis by γ phage and a two-component DFA assay, using cell wall (CW-DFA) and capsule (CAP-DFA) antigens (*11*). The two-component DFA assay is a sensitive, specific, and rapid confirmatory test for *B. anthracis* in cultures (*15*,*16*). The lysis by γ phage (*17*) is highly specific for *B. anthracis,* and when demonstrated concomitantly with the presence of a capsule, confirms the identification. The New York City Department of Health protocol reports a sample as positive only if it has all the following phenotypes: nonmotile, penicillin sensitive, γ-phage positive, and positive by both cell wall and CAP-DFA assays (*11*).

The level-C laboratory has the capacity of the level-B laboratory, plus antimicrobial susceptibility testing and advanced detection methods. It also can help with surge capacity and has much greater biosafety-level working capacity. Advanced detection methods include time-resolved fluorescence and polymerase chain reaction (PCR) (*14*,*18*). These methods are employed to quickly yield preliminary data in advance of the classical microbiology final report (*11*).

The level-D laboratory has the highest level of containment (biosafety level) and expertise in diagnosis. Various tests to determine the molecular characteristics of isolates are conducted, including molecular subtyping with multi-locus variable-number tandem repeat analysis and sequencing of genes coding for 16S ribosomal RNA (*19*,*20*). The analysis allows for identification of a particular pattern that can be associated with geographic, temporal, or other relevant epidemiologic designations. The Centers for Disease Control and the U. S. Army Medical Research Institute of Infectious Diseases maintain level-D laboratories.

Once the *Bacillus* colonies from our patient were identified as catalase positive, nonhemolytic, nonmotile gram-positive rods, the organism was transported to the New York City Department of Health laboratory for further testing, as mandated by LRN. Although the patient’s symptoms did not correlate with classic anthrax disease, a fatal case of inhalational anthrax mimicking intraabdominal sepsis had been recently reported (*21*). The organism isolated in our patient was identified as *B. megaterium*, a frequent blood culture contaminant but rare cause of meningitis, brain abscess, and catheter-related bacteremia. The patient’s strain showed a positive reaction to the CAP-DFA assay. A recent study (*16*) also reported one *B. megaterium* strain (out of 11 strains) with a positive reaction to the CAP-DFA assay. This study analyzed a total of 230 *B. anthracis* isolates; 228 and 229 were positive by CW-DFA and CAP-DFA assays, respectively. A total of 56 *B*. non-*anthracis* strains were also tested; 10 *B. cereus* and 2 *B. thuringiensis* were positive by the CW-DFA assay, and 1 *B. megaterium* strain was positive by CAP-DFA. Analysis of the combined DFA results identified 227 of 230 *B. anthracis* isolates; all 56 strains of the other *Bacillus* species were negative (*16*).

A nonhemolytic, nonmotile *Bacillus* should be highly suspicious for *B. anthracis*. However, species like *B. cereus* subsp. *mycoides, B. megaterium*, *B. firmus*, and *B. circulans* can also be nonhemolytic and nonmotile. The community laboratory is limited in differentiating these species, which can lead to delays in diagnosis and response to potential terrorist events. This case emphasizes the need for local (level A) laboratories to increase their potential to differentiate nonmotile, nonhemolytic *Bacillus* in order to secure a rapid preliminary diagnosis and avoid unnecessary costly treatment. The combined DFA assay would be a potential solution. It provides sensitive and specific confirmation of *B*. *anthracis* cultures within 3 to 6 hours. The assay specificity is similar to the highest levels achieved by PCR assays, and its sensitivity is similar to that of culture or perhaps considerably greater if the patient is receiving antimicrobial agents (*16*).

Dr. Dib is a fellow in training in hematology/oncology at the University of Rochester-Strong Memorial Hospital in New York. His research interests include the relationship of infectious agents and carcinogenesis.
